# Getting in and Staying Alive: Role for Coronin 1 in the Survival of Pathogenic Mycobacteria and Naïve T Cells

**DOI:** 10.3389/fimmu.2018.01592

**Published:** 2018-07-10

**Authors:** Mayumi Mori, Jean Pieters

**Affiliations:** Biozentrum, University of Basel, Basel, Switzerland

**Keywords:** coronin 1, macrophages, *Mycobacterium tuberculosis*, naïve T cell homeostasis, interleukin 7, T cell receptor

## Abstract

There are many different pathogenic stimuli that are able to activate the immune system, ranging from microbes that include bacteria, viruses, fungi, and parasites to host-derived triggers such as autoantigens that can induce autoimmunity as well as neoantigens involved in tumorigenesis. One of the key interactions shaping immunity toward these triggers involves the encounter of antigen-processing and -presenting cells such as macrophages and dendritic cells with T cells, resulting in immune responses that are highly selective for the antigenic trigger. Research over the past few years has implicated members of the coronin protein family, in particular coronin 1, in responses against several pathogenic triggers. While coronin 1 was initially described as a host factor allowing the intracellular survival of the pathogen *Mycobacterium tuberculosis*, subsequent work showed it to be a crucial factor for naïve T cell homeostasis. The activity of coronin 1 in allowing the intracellular survival of pathogenic mycobacteria is relatively well characterized, involving the activation of the Ca^2+^/calcineurin pathway, while coronin 1’s role in modulating naïve T cell homeostasis remains more enigmatic. In this mini review, we discuss the knowledge on the role for coronin 1 in immune cell functioning and provide a number of potential scenarios *via* which coronin 1 may be able to regulate naïve T cell homeostasis.

## Introduction

The vertebrate immune system has evolved to efficiently deal with both intracellular and extracellular pathogens to ensure a battery of defense strategies, both through innate and adaptive mechanisms. The innate immune defense arm can react rapidly as a result of the recruitment of neutrophils, natural killer cells, dendritic cells, and macrophages to the site of infection. These cells not only ensure the direct elimination of the pathogens but also aid in the activation of adaptive immunity by inducing the proliferation, maturation, and expansion of B and T lymphocytes. The concerted action of innate and adaptive immune cells results in an effective clearance of microbial and parasitic pathogens; however, several pathogens have evolved to withstand such immune detection, sometimes by hijacking the immune system at several levels.

For many bacterial pathogens, the initial and often fatal encounter is their interaction with macrophages. These cells are the scavengers of the vertebrate immune system, and typically digest any microbe following their internalization through phagocytosis and delivery to lysosomes and/or autophagosomes (1, 2). Following digestion, pathogen-derived antigens (peptides, lipids, and metabolites) can be bound to the so-called antigen-presenting molecules of the major histocompatibility complex (MHC) class I and class II, cluster of differentiation 1 (CD1), or MHC class I-related protein MR1 complexes, which are then re-routed to the plasma membrane where these antigens can be presented to T lymphocytes. This interaction between antigen-presenting cells and T cells subsequently triggers T cell proliferation/expansion in an antigen-specific manner (3–6). One particularly notorious pathogen, *Mycobacterium tuberculosis*, which is transmitted through aerosols and is phagocytosed by alveolar macrophages, has evolved to hijack this process of intracellular degradation, thereby converting the normal hostile environment of the macrophage into a safe haven. *M. tuberculosis* does so using multiple strategies, including the attenuation of macrophage inflammatory signaling cascades, neutralization of reactive oxygen and nitrogen species, as well as altering its metabolic state (7–10). As a result, instead of being degraded within macrophages, *M. tuberculosis* manages to survive for a prolonged time within these cells, often in a so-called dormant state, and can become reactivated when the health of an infected person deteriorates, for example, following food deprivation or inflammatory stress, such as co-infection by HIV. Apart from its ability to survive within macrophage phagosomes, *M. tuberculosis* has been reported to be released into the macrophage cytosol, where it can activate a number of mechanisms leading to cell death, allowing the dissemination of the bacilli to neighboring cells (11). The capacity of *M. tuberculosis* to withstand intracellular delivery to lysosomes and degradation was initially realized from electron micrographs of *M. tuberculosis*-infected macrophages (12) and has been widely recognized as a major strategy employed by *M. tuberculosis* to establish long-term infections.

## Identification of Coronin 1 as a Survival Factor for Intracellularly Residing Mycobacteria

Given the central importance for *M. tuberculosis* to prevent intracellular delivery to lysosomes for the establishment of a long-term infection, it is not surprising that mycobacteria utilize a number of different strategies to achieve this (8, 9, 13). One of these strategies was found to be the recruitment and retention of a host protein, coronin 1 (also known as P57 or TACO, for Tryptophan Aspartate containing Coat protein), to the cytosolic side of the mycobacterial phagosome. Coronin 1 is expressed in all hematopoietic cell types and is a member of the widely conserved coronin protein family, members of which are expressed in virtually all eukaryotic species (14, 15). The recruitment of coronin 1 activates the calcium/calcineurin pathway that was shown to block phagosome–lysosome fusion and the degradation of the internalized mycobacteria (16, 17). The precise mechanism *via* which calcineurin, a ubiquitously expressed phosphatase, modulates intracellular mycobacterial survival remains to be identified, and it is possible that calcineurin acts in concert with several of the other factors that have been identified to allow *M. tuberculosis* survival within macrophages, including kinases, lipids, metabolites, and signaling molecules (18–23). Coronin 1-dependent modulation of lysosomal trafficking appears to be specific for mycobacteria, since several other types of cargo are readily delivered to lysosomes in a coronin 1-independent manner (16).

The role for coronin 1 in protecting *M. tuberculosis* from intracellular death within macrophages was corroborated by analyzing mice that lack the gene coding coronin 1 (*coro1a*). In macrophages derived from these mice, mycobacteria are readily transferred to lysosomes followed by their destruction (16). However, other than the inability of coronin 1-deficient macrophages to support the intracellular survival of pathogenic mycobacteria, macrophages devoid of coronin 1 appear to be fully functional in terms of phagocytosis, endocytosis, motility, membrane ruffling, and migration (16, 24). This is also notable because coronin family members have been widely implicated in regulation of the F-actin cytoskeleton (25). The reasons for the absence of F-actin-dependent phenotypes in macrophages devoid of coronin 1 may lie within (i) the fact that other coronin family members with redundant roles are co-expressed in macrophages, (ii) a function for coronin 1 upstream of F-actin modulation, or (iii) differences in experimental protocols used to analyze coronin 1’s function in macrophages. Interestingly, upon macrophage activation as occurs during an inflammatory stimulus, coronin 1 functions to switch the mode of uptake from phagocytosis to macropinocytosis, thereby enabling macrophages to rapidly internalize large amount of cargo and shuttling these to lysosomes for degradation (26, 27). Thus, it appears that *M. tuberculosis*, perhaps in the course of its long-term co-evolution with their mammalian hosts, has gained the capacity to utilize coronin 1-dependent arrest of phagosome–lysosome fusion to allow long-term survival within macrophages, that are precisely those cells destined to destroy any incoming bacilli.

## Peripheral T Cell Survival and Coronin 1

The availability of mice lacking coronin 1 also allowed the analysis of other hematopoietic cell types with respect to their dependence on coronin 1 for proper functioning. Strikingly, whereas virtually all other cell types appear to be unaffected by the absence of coronin 1, there is one notable exception: mice devoid of coronin 1 are profoundly deficient in T cells (28–31). Interestingly, this T cell deficiency is exclusively found in peripheral lymphoid organs: T cell development and selection, as for example occurring in the thymus, is not affected by the absence of coronin 1 (32). Several explanations have been put forward to explain the peripheral T cell deficiency in mice lacking coronin 1: first, the above-mentioned role for coronin proteins in modulating F-actin was suggested to be responsible for inducing T cell death, *via* a proposed role for coronin 1 in reducing F-actin levels, in the absence of which elevated F-actin may act to induce cell death (28). However, subsequent work showed that in leukocytes coronin 1 does not modulate F-actin, and furthermore that accumulation of F-actin does not correlate with the induction of cell death (30, 33). Alternatively, coronin 1 may be involved in the transduction of signals downstream of the T cell receptor (TCR), in the absence of which pro-apoptotic, rather than pro-survival signals, are being activated (29, 30, 33). Such a pro-survival role for coronin 1 must be selective for peripheral naïve T cells, since both thymic selection and effector/memory T cells do not depend on coronin 1 for either survival or functionality (29, 32).

## Homeostatic Control of Peripheral Naïve T Cell Numbers

As mentioned above, while peripheral CD4 and CD8 positive T cells are profoundly depleted upon coronin 1 inactivation, T cell development and selection in bone marrow and thymus is virtually undisturbed. This is a surprising observation since two of the main drivers of naïve T cell homeostasis, namely, MHC:TCR signaling and interleukin (IL)-7:IL-7 receptor (IL7R) signaling are both important for thymic T-cell survival (34). Thus, either these signaling pathways require coronin 1 exclusively in peripheral lymphoid organs or, alternatively, coronin 1 is involved in an as yet undefined pathway responsible for peripheral naïve T cell survival.

Homeostatic proliferation and survival are differently controlled between naïve and memory T cells and between CD4 and CD8 T cells (35). For the discussion here, we focus on the naïve CD4 T cell subset, which is most severely suppressed in coronin 1-deficient mice. The mechanisms that have been suggested to maintain naïve CD4 T cells include, besides the aforementioned IL-7 signaling and MHC–TCR interaction, other signaling pathways such as those involving IL-2, 15, and type I interferons, although these appear to be involved to a lesser extent (34, 36–38).

Interleukin-7 has a central role in early lymphopoiesis in the thymus to drive the selection of CD8 lineage-committed cells (39–41). IL-7 does so, *via* activation of the IL7R pathway, through induction of the expression of the pro-survival factor Bcl2 and inhibiting the pro-apoptotic factors Bad and Bax. Regarding the role for IL-7 on maintenance of the peripheral naïve CD4 T cell pool, there are conflicting data in the literature. In support of a role for IL-7 in naïve CD4 T cell survival, Tan et al. demonstrated a failure of transferred T cells to survive when adoptively transferred to IL-7-deficient mice (42). Also, overexpression of IL-7 was shown to enhance T cell proliferation in a lymphopenic mouse model (43). Furthermore, several studies documented enhanced peripheral T cell proliferation upon overexpression of IL-7 or the IL7R (44, 45), although this was not observed in all animal models (46). Furthermore, *in vivo* infusion of IL-7 results in increased naïve CD4 T cell numbers, although the effect on CD8 T cells is many-fold higher (47–49). On the other hand, a number of studies have reported that IL-7 is dispensable for CD4 T cell proliferation and survival; for example, blockade of the IL-7 receptor alpha chain (IL-7Rα) inhibits only a minor population of low-rate proliferating naïve CD4 T cells after transfer to RAG2-deficient recipients, without affecting the high-rate proliferating cells (50). Also, while administration of anti-IL-7 antibodies reduces the survival of peripheral CD4 positive T cell numbers (51), it does so only when IL-4 is also depleted (52), suggesting redundant roles for cytokines sharing the common receptor gamma chain (γ_c_). Conversely, more recent work using a xenogeneic model suggests that increasing IL-7 signaling does not affect peripheral T cell numbers while it does modulate T cell development in the thymus (53). Furthermore, conditional genetic deletion of IL-7Rα or γ_c_ at the late-stage of thymic development, circumventing the suppressive effect on early lymphopoiesis, showed only minimal reduction in CD4 single positive thymocytes, compared with a profound reduction of CD8 single positive T cells, whereas peripheral naïve CD4 T cell numbers have not been addressed in these studies (54, 55). Finally, it has been proposed that rather than a direct availability of IL-7 to CD4 T cells, IL-7 signaling on antigen-presenting cells may be the main driver of homeostatic proliferation of naïve CD4 T cells *in vivo* (56, 57).

Thus, while the role for IL-7 in CD8 T cell lineage selection in the thymus is clearly established, it appears to be dispensable for CD4 T cell lineage selection, and an exclusive role for this cytokine in the maintenance of peripheral naïve CD4 T cell survival is unclear. Perhaps, IL-7 is mainly required for depletion-induced (“homeostatic”) proliferation rather than maintenance of T cell numbers under non-perturbed situations. Given the normal thymic T cell development observed in mice lacking coronin 1 (32), it is unlikely that coronin 1 plays a role directly downstream of IL-7 signaling. However, it is possible that coronin 1 works in concert with IL-7 signaling to allow peripheral naïve CD4 T cell survival.

The second trigger that is widely reported to be involved in naïve CD4 T cell survival is TCR signaling by MHC:peptide complexes. While, similar to IL-7 signaling, MHC molecules play a crucial role during thymic selection (58, 59), several studies have reported that MHC molecules are important for peripheral T cell survival and proliferation (60–62). In particular, peptides loaded on MHC, possibly self-ligands, were considered to be crucial for homeostatic expansion of CD4 T cells, as shown by reduced expansion in hosts that lack peptide presentation on MHCII (63, 64), suggesting that specific TCR–MHC interaction is important.

Besides the aforementioned studies that report a role for MHC molecules in the survival and proliferation of naïve T cells, there are several studies suggesting that naïve T cell survival is MHC independent. For example, both survival and proliferation of peripheral CD4 T cells have been reported to occur in the absence of MHC class II molecules (65, 66). In addition, another study indicates that MHC may be important for proliferation but not for survival of naïve CD4 T cells (67), considering also the long half-life of T cells after depletion of MHC class II molecules (68, 69).

Regarding the role for peptide presentation on MHC class II molecules, one study using the same H2M-deficient system as Viret et al. (63) have shown dispensability of peptide ligands for peripheral T cell survival (68). Moreover, DC–T cell synapse accompanied with polarized PKCθ phosphorylation, indicating the existence of TCR signaling, was detected without antigens or MHC itself (66). Thus, T cell responses supposed to be important for peripheral T cell survival and homeostatic proliferation may not require interaction with cognate peptide–MHC complexes.

As described above, arguments have been brought forward both in favor of and against a role for MHC class II molecules in naïve T cell survival. What exactly underlies the discrepancy between these opposing results, that in part were obtained using the same experimental model [for example, the same H2M-deficient mouse was used to conclude for and against a role of MHC class II in T cell survival, see Ref. (63, 68)], remains to be analyzed and could lie within an inability to distinguish survival and proliferation, the usage of mice deficient in different components of MHC, or the subtype of peripheral T cells analyzed (e.g., naïve versus memory) (70–72). Interestingly, even in the complete absence of MHC class II molecules in both mice and man, peripheral naïve T cells can be maintained, even for prolonged times (72–74).

Thus, the extent to which MHC–peptide:TCR interaction is important for peripheral naïve CD4 T cell survival remains unclear. Given the normal thymic development of T cell precursors in mice deficient in coronin 1 and the important role for MHC–TCR signaling in that process, it is unlikely that coronin 1 plays a prominent role in MHC-dependent T cell activation to generate mature T cells. Whether the role for coronin 1 in the maintenance of peripheral T cells involves intracellular events downstream of TCR remains to be analyzed (29). Coronin 1 has been suggested to act through activation of calcium/calcineurin signaling and through modulation of the cytoskeleton (16, 28–31, 33), but how, exactly, defects in these pathways would result in such a selective phenotype (peripheral naïve T cell deficiency) remains unclear. It should also be mentioned that little is known about the molecular mechanisms underlying the transition from semi-mature single positive thymocytes to mature naïve T cells in the periphery (75). Future work exploring a possible role for coronin 1 in both the above described IL-7 and MHC–TCR signaling as well as yet unexplored pathways may allow to shed light not only on the molecular mechanisms in which coronin 1 is involved but also possibly contribute to a better understanding of peripheral naïve T cell homeostasis.

## Author Contributions

MM and JP conceived and wrote the paper.

## Conflict of Interest Statement

The authors declare that the research was conducted in the absence of any commercial or financial relationships that could be construed as a potential conflict of interest.
